# Electroacupuncture for lumbar disc herniation: A protocol for systematic review and meta-analysis

**DOI:** 10.1097/MD.0000000000019867

**Published:** 2020-04-24

**Authors:** Yuquan Shen, Qun Zhou, Leixiao Zhang, Liang Gao, Di Zhang, Xinling Wang, Yang Yu, Zhengsong Zhang, Jianjia Liu, Shumi Liang, Guilong Zhang

**Affiliations:** a Affiliated to The First People's Hospital of Longquanyi District, Chengdu, Sichuan; b Chengdu University of Traditional Chinese Medicine; c Acupuncture and Tuina School, Chengdu University of Traditional Chinese Medicine, Chengdu, Sichuan; d Boai hospital affiliated to China rehabilitation research center, Beijing; e Hospital of Chengdu University of Traditional Chinese Medicine, Chengdu, Sichuan, China.

**Keywords:** electroacupuncture, lumbar disc herniation, protocol, systematic review

## Abstract

**Background:**

This systematic review aims to evaluate the effectiveness of electroacupuncture in treatment of lumbar disc herniation (LDH).

**Methods:**

Electronic databases of all electroacupuncture for LDH will be searched at PubMed, Cochrane Library, Springer, EMBASE, China National Knowledge Infrastructure (CNKI), Wan-Fang, and Chinese Biological Medical disc, (CBM) from inception to February 29, 2020, with language restricted in Chinese and English. The primary outcome is Japanese Orthopedic Association Scores, a quantification scale for a comprehensive assessment according to patients’ subjects feeling and objective function. Secondary outcomes included visual analogue scale (VAS), Oswestry dysfunction index (ODI), Pittsburgh sleep quality index (PSQI), Self-rating anxiety scale (SAS), self-depression rating scale (SDS), follow-up relapse rate. The systematic review and searches for randomized controlled trials of this therapy for LDH. The Cochrane RevMan V5.3 bias assessment tool is implemented to assess bias risk, data integration risk, meta-analysis risk, and subgroup analysis risk (if conditions are met). Mean difference (MD), standard mean deviation (SMD) and binary data will be used to represent continuous results.

**Results:**

This study will provide a comprehensive review and evaluation of the available evidence for the treatment of LDH with this therapy.

**Conclusion:**

This study will provide new evidence to evaluate the effectiveness and side effects of electroacupuncture for LDH. Due to the data is not personalized, no formal ethical approval is required.

## 1 Introduction

### 1.1 Description of the condition

Lumbar disc herniation (LDH) refers to a pathological condition as ruptured anulus fibrous or nucleus pulposus beyond the intervertebral disc space due to degeneration disc and exterior pressure.^[1–3]^ The displacement of disc stimulates the nerve root and contributes the most common cause of sciatica and chronic low back pain, affecting nearly 1% to 5% of population annually.^[4]^ Ever since it occurred, degeneration disc makes LDH easy to be recurrent. LDH has bad influence on people's daily life, work and lifestyle, even leads to long term depression, chronic neurofunction deficit and disability.^[5]^ In recent years, the incidence of LDH has been rising and the onset age has inclined to be younger.^[6]^ The recurrence and complication place a heavy enough burden on both individual and the society. It occurs among those working adults whose age are most under 50 years old and has become a matter of concern about worrying occupational health issue because of the affection of continuation and resumption of employment.^[7]^

The common signs and symptoms of LDH are low back pain, radicular leg pain, abnormal sensation, degenerated strength of low limber, cauda equina syndrome, and intermittent claudication. Patients reported the primary signs and symptoms are low back pain and radicular leg pain which are also the most suffering throughout the whole process of LDH.^[8–9]^ Etiology factors have been investigated in an attempt to understand how LDH produces the symptoms. The prim contributor is degenerative changes of intervertebral disc which is found to increase the press of disc and compresses the nerve root. Inflammation also plays a significant role on the etiology factors which is known to lead to pain. Nucleus pulposus is variety of sequestered antigen, as the herniation occurs, it will be exposed to immune system and leads to immune respond and results in inflammatory pain.^[10–11]^ Concentration of varieties of chemical, immune and neurogenic inflammatory factors including prostaglandin E2, NO, matrix metalloproteinases, calcitonin generelated peptide, are shown to be higher in the patients with LDH than those of non-herniation controls.^[12–14]^ Apoptosis mechanism is another contributor according to previous research. Surveys show there are a large number of apoptotic chondrocytes in degenerative lumbar disc tissue, which leads to metabolic disturbance of disc matrix, and makes further promotion of disc degeneration in turn.^[15–17]^

LDH has forged close ties with anxiety and depression. Pain-related anxiety has a high rate among patients with LDH, it is reported the incidence ranges from 10.6 to 57.4%.^[18]^ Mccracken has found potential and persistent pain stimulates specific tissues and organs, such as the reaction of muscles and nerves, influencing the thinking ability and judgment which makes patients more anxious about pain and trapped in vicious circle.^[19]^ Another research shows that the degree and duration of pain determines anxiety level.^[20]^ Patients are more likely to be sensitive to social stress under both anxiety and pain. The pain-related anxiety has a serious impact on daily life and harms more than the effect the pain itself brings up. It is recognized conclusion that depression is the common psychological symptom of LDH due to the long-term pain. A survey has revealed the incidence of depression in patients with chronic pain ranges from 17.8% to 92.4%.^[21]^ According to the theory of “chronic pain-psychological disorder-chronic pain exacerbation- psychological disorder exacerbation”, patients may have fear of the chronic pain and trapped in the vicious circle.^[22]^ These impacts may cause the patients lose faith of life or even eschew medical treatment. Pain is the leading cause of the psychological changes in patients with LDH and it has mutual influence with anxiety, depression, and fear. Chronic pain leads to negative emotions relieving pain is very important in the treatment of LDH.

Interventions for LDH are aimed at relieving pain and improving mobility, function and life qualify. The first-line choices for LDH are non-operative treatments which may be consisted of anti-inflammatory medications, physical therapy, education and epidural steroid injection, and others.^[23]^ There is no consensus to give a recommendation for non-operative or surgical interventions, apart from some cases, symptomatic patients are advised to take consider of surgical treatment if they are not resistant to the severe sciatica or local lumbago.^[24]^ To relieve the pain, surgical treatments performed faster than non-operative ones. However, surgery results in no benefit over a mid-term or long-term follow-ups.^[25–26]^ Due to the degeneration of lumbar intervertebral disc, recurrent herniation occurs and many patients seek for reoperation following primary surgery.^[27–28]^ In consideration of the high recurrent rate, most clinic doctors advise patients to accept non-operative treatments if they do not need to take an immediate surgery. Non-operative treatments are, therefore, widely used for LDH. There are numerous non-operative treatments but also a lack of satisfactory for both doctor and patients. Each therapy has advantages and disadvantages. Currently, in addition to those non-operative treatments referred above, some complementary and alternative therapies drew more and more attention to treat LDH.

As one of the complementary and alternative therapies, Traditional Chinese medicine (TCM) therapies play a vital role in preventing and treating many varieties of diseases including LDH. As one of TCM therapies, acupuncture has aroused lots of attraction from all over the world due to its efficiency in the clinical treatment.^[29]^

### 1.2 Description of the intervention

Acupuncture is one of the key components of TCM therapies in which thin needles are inserted into the body. It is developed and practiced in ancient China dating from Shang Dynasty (1600–1100 BC). Acupuncture is based on TCM theory in which health is deemed as keeping balance of energy that is called *Qi* flowing a circulation through the channels and meridians in the body. Acupuncture corrects the imbalance of the flowing of *Qi* via a needle inserted into special acupoints along the meridians.^[30]^ Acupuncture has been proved to be efficient in China for more than 2000 years and it had been widely used in treatment of varieties of diseases. Due to the efficiency of pain relief, it has increasingly drawn widespread attention and it was recommended by the World Health Organization since 1980 as an alternative therapy for 43 different disorders. An American survey found that 8 million Americans have been accepted acupuncture therapy in their lifetime and the most common used is for low back pain.^[31–32]^ According to recent studies, acupuncture can relieve not only pain but also the other symptoms including improving the functional recovery of the whole body. Electroacupuncture is based on acupuncture where a kind of chosen small current passed between pairs of acupuncture needles. The same acupoints are inserted with needles and several pairs of needles can be simultaneously stimulated during the treatment. With the standard operation, electroacupuncture is safe to keep current under control and not beyond the endurance of patients.

### 1.3 How the intervention might work?

According to TCM theory, LDH is caused by *Qi* stagnation and blood stasis, external trauma or deficiency of *Qi* and blood, which all result in obstruction of *Qi* and blood flowing in the meridians. The principle of TCM treatment is to promote the circulation of *Qi* and blood of which relieving pain, activating the *Qi* stagnation and removing blood stasis, improving function. Electroacupuncture corrects the imbalance of the meridians through regulating *Qi* and blood prolonged stimulation on the acupoints from special devices. Some modern medicine surveys found that LDH is treated by electroacupuncture may be through improving local microcirculation, adjusting the abnormal ultra-structure of nerve cell, regulating the immune system, decreasing the level of inflammatory cytokines, influencing the speed of nerve conduction velocity.^[33–37]^ Even there are many studies about how electroacupuncture works on LDH and some researchers came up with a variety of hypothesis but no consensus has been reached, leaving the main mechanism still remaining unclear.

### 1.4 Objectives

To develop treatment recommendations, we systematically evaluated the efficacy and safety of electroacupuncture for LDH.

## 2 Methods

### 2.1 Study registration

PROSPERO registration number is CRD42020151213. This protocol report was performed according to the Preferred Reporting Items for Systematic Reviews and Meta-Analyses Protocols (PRISMA-P) statement guidelines.^[38]^ The review will be conducted in accordance with the PRISMA guidelines and follows the recommendations of the Cochrane Handbook for Systematic Reviews of Interventions.^[39–40]^ If we refine the procedures described in this protocol, we will update the record in the PROSPERO and disclose them in future publications related to this study.

### 2.2 Inclusion criteria for study selection

#### 2.2.1 Types of study

In order to evaluate the efficacy of electroacupuncture in the treatment of LDH, this paper only reviewed the randomized controlled trial (RCT) between electroacupuncture and the control group, including drug therapy, no treatment, placebo, diet and physical therapy, etc. In addition, both Chinese and English publications are restricted languages to this review. All RCT that are not subject to publication state constraints will be included. If the experiment shows that the phrase is random and the blind method is not restricted, it will be regarded as a random study. Animal mechanism studies, case reports, self-controlled, non-randomized controlled trials, random crossover studies, and quasi-randomized trials will be excluded.

#### 2.2.2 Types of participants

Regardless of gender, age, ethnicity, education and economic status, patients with LDH who meet the diagnostic criteria.^[41–43]^

#### 2.2.3 Types of intervention

The experimental group should be treated with electroacupuncture and acupoints used according to TCM nomenclature. The types of seed used and the duration of treatment will be unlimited. Electroacupuncture combined with other conventional therapy should be excluded.

The following treatment comparisons will be investigated:

1. Electroacupuncture compared with no treatment;
2. Electroacupuncture compared with placebo or sham treatment;
3. Electroacupuncture compared with other active therapies;
4. Electroacupuncture in addition to active therapy compared with the same active therapy.

We will assess and compare the electroacupuncture according to how the acupuncturists have been trained and educated, on their clinical experience, on total numbers of electroacupuncture sessions, and on the treatment duration and frequency, etc.

#### 2.2.4 Types of outcome measures

The primary outcome of this review was body function which is preferentially extracted from Japanese Orthopedic Association Scores. Secondary outcomes include visual analogue scale (VAS), Oswestry disability index (ODI), self-rating anxiety scale (SAS), self-rating depression scale (SDS), Pittsburgh sleep quality index (PSQI), recurrence rate during the follow-up period, and adverse events.^[44–46]^ The system review will be performed independently.

### 2.3 Data sources

Our systematic review will search all RCTs of electroacupuncture for LDH electronically, regardless of publication status and language, by February 29, 2020. Databases include: PubMed, EMBASE, Springer, Web of Science, Cochrane Library, WHO International Clinical Trials Registry Platform (ICTRP), Traditional Chinese Medicine databases (TCMD), China National Knowledge Infrastructure (CNKI), China Biomedical Literature Database (CBM), Chinese Scientific Journal Database (VIP) and Wan-Fang database. Other sources, including reference lists of identified publications and meeting minutes, will also be searched.

### 2.4 Search strategy

The search strategy will be followed the PRISMA guidelines. The key search terms are (“chronic low back pain” OR “back pain” OR “lumbar pain” OR “lumbago” OR “leg pain” OR “sciatica” OR “low limb pain”) AND (“electroacupuncture” OR “traditional Chinese therapy of electroacupuncture” OR “complementary therapy of electroacupuncture”) AND (“randomized”). The search strategy will be adapted to different databases demands. Search strategy in PubMed is shown in Table 1.

**Table 1:**
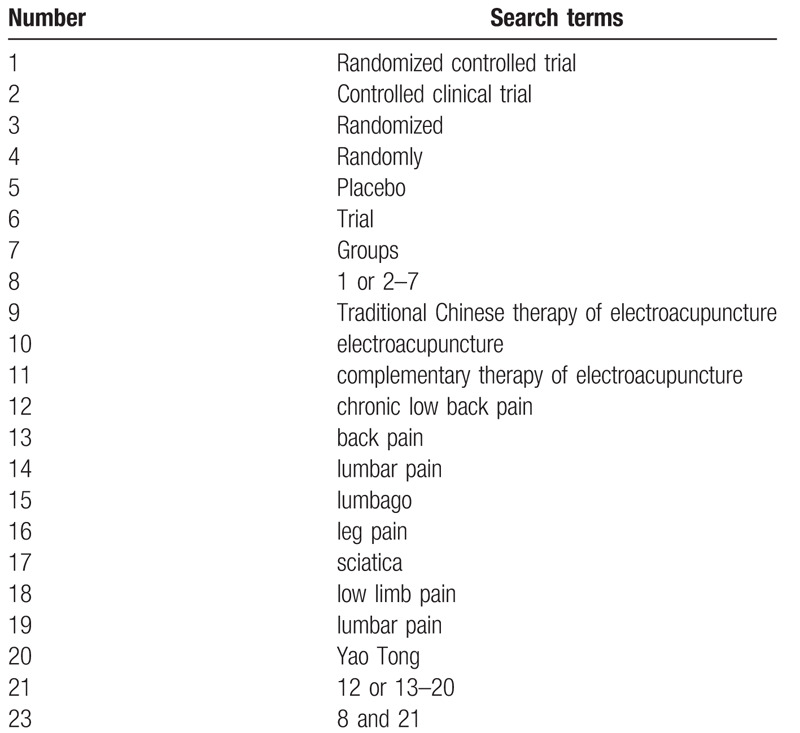
PubMed search strategy.

### 2.5 Data collection and analysis

#### 2.5.1 Selection of studies

**Figure 1:**
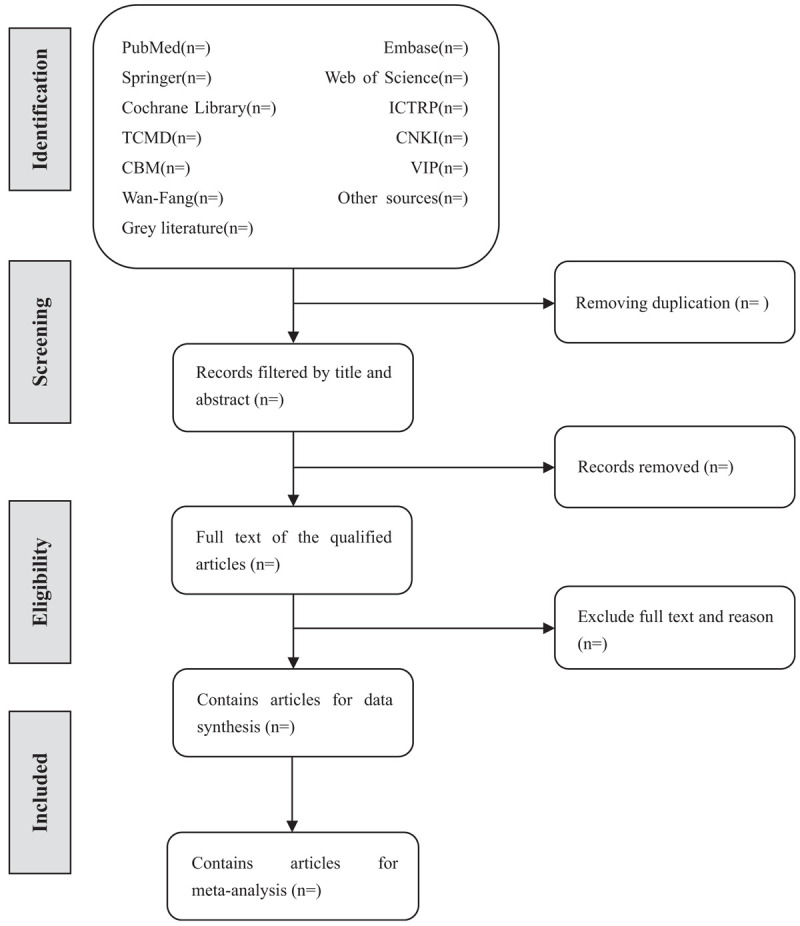
Flow diagram of studies identified.

Prior to literature retrieval, all reviewers are trained to ensure a basic understanding of the background and purpose of the review. In the literature screening process, we will use EndNote software (V.X8) document management software. The 2 comment author (GLZ and LG) will be in strict accordance with the inclusion criteria, independent screen all retrieval research, read the title, abstract and key words in the literature, and determine which meet the inclusion criteria. We will obtain the full text of all relevant studies for further evaluation. Excluded studies will be documented and explained. If there is a disagreement in the selection process, it will be discussed by the 2 authors (GLZ and LG) and the third author (LXZ) and arbitrated if necessary. If necessary, we will contact the trial author for clarification. The primary selection process is shown in a PRISMA flow chart (Fig. 1).

#### 2.5.2 Data extraction and management

The authors will extract data independently from the selected report or study and fill out the data extraction form. We will obtain data on general information, participants, methods, interventions, outcomes, results, adverse events, conflicts of interest, ethical recognition, and other information. For publications with insufficient or ambiguous data, we will attempt to obtain information from the corresponding authors by e-mail or telephone. Any differences will be resolved through discussions between the 2 authors, and further differences will be arbitrated by the third author (LXZ).

#### 2.5.3 Assessment of risk of bias and reporting of study quality

The authors (QZ and YY) will use the Cochrane Collaboration's bias risk assessment tool to assess the risk of bias in all included studies. We will assess the risk of bias in the following areas: sequence generation, assignment sequence hiding, the blindness of participants and staff, and result evaluators, incomplete outcome data, selective outcome reporting, and other sources of bias. This review uses L, U, and H as the key to these assessments, where L (low) indicates a lower risk of bias, U (unclear) indicates that the risk of bias is uncertain, and H (high) indicates a higher risk of bias. If inconsistent results appear, the final decisions will be made by the third author (LXZ). Information on the risk of biased assessments included in the study is summarized in tabular form and the results and impacts are critically discussed. If the information is ambiguous, we will try to contact the author. For repeated publications, we only select the original text.

#### 2.5.4 Measures of treatment effect

Data analysis and quantitative data synthesis will be performed using RevMan V.5.3. For continuous data, if there is no heterogeneity, we will use mean difference (MD) or standard MD (SMD) to measure the therapeutic effect of 95% confidence interval (CI). If significant heterogeneity is found, a random effects model will be used. For dichotomous data, we will use the 95% CI risk ratio (RR) for analysis.

#### 2.5.5 Unit of analysis issues

We will include data from parallel group design studies for meta-analysis. Only the first phase of the data will be included in the random crossover trial. In these trials, participants were randomly divided into 2 intervention groups and individual measurements for each outcome of each participant were collected and analyzed.

#### 2.5.6 Management of missing data

If the primary result has missing or incomplete data, we will contact the author of the communication to obtain the missing data. If it is never available, exclude the experiment from the analysis.

#### 2.5.7 Assessment of heterogeneity

We will use the Review Manager to assess efficacy and publication bias (version 5.3, Nordic Cochrane Centre, Copenhagen, Denmark). The forest map is used to illustrate the relative strength of the effect. The funnel plot is used to illustrate the bias because the number of trials exceeds 10. If a significant difference is detected, a random effects model will be used.

#### 2.5.8 Assessment of reporting biases

We will use a funnel plot to detect report bias. If more than 10 trials are included, the funnel plot will be used to assess the reported bias. If the funnel plot is found to be asymmetrical, analyze the cause using Egger method. We will include all eligible trials regardless of the quality of the method.

#### 2.5.9 Data synthesis

We will use RevMan for all statistical analysis. If considerable heterogeneity is observed, a 95% CI random effects model will be used to analyze the combined effect estimates. Subgroup analysis will be performed with careful consideration of each subgroup if necessary.

#### 2.5.10 Subgroup analysis

There is no presubgroup plan. Subgroup analysis was performed based on control interventions and different outcomes.

#### 2.5.11 Sensitivity analysis

Based on sample size, heterogeneity quality, and statistical models (random or fixed-effect models), we will perform sensitivity analysis.

#### 2.5.12 Grading the quality of evidence

The quality of evidence for all outcomes will be judged by the Grading of Recommendations Assessment, Development, and Evaluation (GRADE) working group approach. Bias risk, consistency, directness, precision, publication bias, are aspects of our assessment. High, medium, low or very low represents the 4 levels of evaluation.

## 3 Discussion

LDH is a common spinal disorder for physician visits in the United States. It is the main contributor to low back pain and sciatica due to the displacement of disc, which affects a large population suffering all around the world. Different forms of non-operative treatments should be taken in consideration as priority unless it is in acute stages such as neurological deficit or unendurable pain. As numerous of studies have proved that electroacupuncture is effective, safe, operable, low-cost and promising for LDH,^[47–50]^ therefore it has been prevalent over the last few decades to reduce the need for surgery.

The evaluation of this systematic review will be divided into 4 parts: identification, the inclusion of literature, data extraction, and comprehensive analysis of data. According to the Cochrane method, this study is based on the analysis of clinical RCT evidence at home and abroad, searching and screening the main electronic literature database with evidence-based medical evidence, providing clinicians with more convincing evidence in decision-making, to better guide clinical treatment.

## Author contributions

**Conceptualization:** Yuquan Shen.

**Methodology:** Xinling Wang, Zhengsong Zhang, Jianjia Liu, Di Zhang

**Software:** Leixiao Zhang, Qun Zhou, Shumi Liang, Yu Yang

**Supervision:** Guilong Zhang,

**Validation:** Yu Yang,

**Writing–original draft:** G. Zhang, Leixiao Zhang, Liang Gao, Yuquan Shen, Yu Yang

Guilong Zhang orcid: 0000-0001-7812-8096.
